# SARS-CoV-2 variant-related abnormalities detected by prenatal MRI: a prospective case–control study

**DOI:** 10.1016/j.lanepe.2023.100587

**Published:** 2023-01-21

**Authors:** Patric Kienast, Daniela Prayer, Julia Binder, Florian Prayer, Sabine Dekan, Eva Langthaler, Benjamin Sigl, Sabine Eichinger, Nicole Perkmann-Nagele, Ingrid Stuempflen, Marlene Stuempflen, Nawa Schirwani, Petra Pateisky, Christian Mitter, Gregor Kasprian

**Affiliations:** a Department of Biomedical Imaging and Image-Guided Therapy, Division of Neuroradiology and Musculoskeletal Radiology, Medical University of Vienna, Vienna, Austria; b Department of Obstetrics and Feto-Maternal Medicine, Medical University of Vienna, Vienna, Austria; c Department of Pathology, Medical University of Vienna, Vienna, Austria; d Department of Medicine I, Division of Hematology and Hemostaseology, Medical University of Vienna, Vienna, Austria; e Department of Laboratory Medicine, Medical University of Vienna, Vienna, Austria; f Department of Obstetrics & Gynecology, Klinik Floridsdorf, Vienna, Austria

**Keywords:** COVID-19, SARS-CoV-2, COVID-19 during pregnancy, Omicron, Variants, Prenatal MRI, Placenta, Vascular

## Abstract

**Background:**

There are known complications for fetuses after infection with SARS-CoV-2 during pregnancy. However, previous studies of SARS-CoV-2 in pregnancy have largely been limited to histopathologic studies of placentas and prenatal studies on the effects of different SARS-CoV-2 variants are scarce to date. To examine the effects of SARS-CoV-2 variants on the placenta and fetus, we investigated fetal and extra-fetal structures using prenatal MRI.

**Methods:**

For this prospective case–control study, two obstetric centers consecutively referred pregnant women for prenatal MRI after confirmed SARS-CoV-2 infection. Thirty-eight prenatal MRI examinations were included after confirmed infection with SARS-CoV-2 and matched 1:1 with 38 control cases with respect to sex, MRI field strength, and gestational age (average deviation 1.76 ± 1.65, median 1.5 days). Where available, the pathohistological examination and vaccination status of the placenta was included in the analysis. In prenatal MRI, the shape and thickness of the placenta, possible lobulation, and vascular lesions were quantified. Fetuses were scanned for organ or brain abnormalities.

**Findings:**

Of the 38 included cases after SARS-CoV-2 infection, 20/38 (52.6%) were infected with pre-Omicron variants and 18/38 (47.4%) with Omicron. Prenatal MRIs were performed on an average of 83 days (±42.9, median 80) days after the first positive PCR test. Both pre-Omicron (*P* = .008) and Omicron (*P* = .016) groups showed abnormalities in form of a globular placenta compared to control cases. In addition, placentas in the pre-Omicron group were significantly thickened (6.35, 95% CI .02–12.65, *P* = .048), and showed significantly more frequent lobules (*P* = .046), and hemorrhages (*P* = .002). Fetal growth restriction (FGR) was observed in 25% (n = 5/20, *P* = .017) in the pre-Omicron group.

**Interpretation:**

SARS-CoV-2 infections in pregnancy can lead to placental lesions based on vascular events, which can be well visualized on prenatal MRI. Pre-Omicron variants cause greater damage than Omicron sub-lineages in this regard.

**Funding:**

10.13039/501100001821Vienna Science and Technology Fund.

**Research in context**

**Evidence before this study**

Although it is now known that infection with SARS-CoV-2 during pregnancy can cause serious complications in mothers and fetuses, very few prenatal studies exist in the literature on possible intrauterine damage to fetal and extrafetal structures. A few histopathologic studies address placental changes after infection but due to the postnatal examination, they can provide only limited information about already prenatally present pathological changes. In addition, the current body of studies on variant-specific changes after SARS-CoV-2 during pregnancy is very sparse. MRI has not been used so far for these purposes. However, MRI is known to provide direct information on the placental structure (while ultrasound assessment of placental function is based mainly on Doppler measurements) and allows to assess the fetal organs in detail.

**Added value of this study**

In this prospective multicentric case–control study that included 76 prenatal MRI scans (38 after SARS-CoV-2 infection and 38 controls), placentas after infection with pre-Omicron and Omicron variants showed a higher frequency of vascular-based lesions associated with fetal impairment, compared to noninfected controls. These were more pronounced with the pre-Omicron variants than with the Omicron sub-lineages. In possible upcoming future variants with similar pathogenetic mechanisms as, for instance, the Delta variant, the placenta should be monitored closely with respect to vascular lesions and potential fetal impairment.

**Implications of all the available evidence**

According to our results some SARS-CoV-2 variants may involve the placenta more frequently than others. Therefore, fetuses from such pregnancies may be under a greater risk of placental-related vascular malperfusion injuries, either in form of fetal growth restriction or vascular lesions in fetal organs.

## Introduction

Pregnant women are more likely to be hospitalized or to require intensive care unit admission when affected by severe acute respiratory syndrome coronavirus type 2 (SARS-CoV-2) infection than non-pregnant women. Moreover, the overall the perinatal mortality after SARS-CoV-2 infection is increased compared to a non-pregnant cohort.^1^^,^^2^ Vertical virus transmission to the fetus is infrequent (0–3.2%),^3^, ^4^, ^5^ suggesting that the placenta serves as an immunologic barrier against SARS-CoV-2.^6^ However, vascular impairment of the placenta has been described histologically in some cases.^7^, ^8^, ^9^, ^10^ The consequences of so-called vascular malperfusion syndromes, such as increased rates of fetal growth restriction (FGR), prelabour rupture of membranes (PROM), and consequently need for delivery by Caesarean section, have been observed at a higher frequency in infected compared to uninfected individuals.^11^ In addition, the overall rate of miscarriage is increased—even with a milder course of disease.^1^^,^^2^

Placental lesions associated with maternal vascular malperfusion (MVM) can be detected postnatally in approximately 1/3 of births at term and in approximately 1/2 of preterm births in all pregnancies. MVM of the placental bed is the most common cause of FGR and is characterized by placental abnormalities of maternal decidual vessels and includes irregular spiral artery remodeling, as well as defective oxygenation or abnormal perfusion in the intervillous space.^12^

Fetal vascular malperfusion (FVM) is an umbrella term for disorders of the fetal placental vasculature caused by blood flow restrictions. Possible pathohistological correlates are villous stromal-vascular karyorrhexis, segmental avascular villi, or occlusive or non-occlusive thrombi.^13^ FVM can be found in 3.5%–8.7% of all placentas.^14^, ^15^, ^16^

Several postnatal histological studies have demonstrated an increased incidence of MVM and FVM following infection with SARS-CoV-2, mainly due to intervillous thrombi and inflammatory processes.^7^, ^8^, ^9^, ^10^ A recent meta-analysis by Suhren et al. failed to detect any COVID-19-specific patterns.^17^ This meta-analysis, however, neither differentiated between SARS- CoV-2 variants, as only several pre-Omicron variants existed at the time of the study, nor included *in-vivo* or *in-utero* imaging data.

To date, all but a single study has relied on postnatal placental changes: Sotiriou et al. evaluated placentas of pregnant COVID-19 patients via prenatal ultrasound and demonstrated placental lesions defined as the formation of placental lakes with perivillous fibrin deposits and subchorionic fibrin deposition in 80% of hospitalized women on ultrasound examination.^18^

The excellent tissue contrast of Magnetic resonance imaging (MRI) allows for the non-invasive identification of specific placental abnormalities *in vivo* before birth, whereas pathological workup can only approach the “end stage” of placental development ex vivo. Structural changes, reported by pathology after delivery can be well detected earlier—at prenatal stages of development—by fetal MRI.

In this study, we took advantage of the excellent diagnostic capabilities of fetal MRI in placental assessment and aimed to test the hypothesis of whether prenatal MRI can detect placental damage after SARS-CoV-2 infection during pregnancy and whether the infection has a possible visualizable impact on fetal development and fetal organs.

## Methods

### Study design and patients

For this prospective case–control study, we examined fetal and extra-fetal structures with MRI of pregnant women, who were consecutively referred to prenatal MRI by two obstetric centers after confirmed SARS-CoV-2 infection.

SARS-CoV-2-variants were determined either by direct sequencing, by real-time RT-PCR melting curve analysis, or by the time of infection. For this purpose, weekly sequencing figures for the province of Vienna provided by the Austrian Agency for Health and Food Safety were consulted,^19^ and cases were considered only if one viral variant exceeded all others combined by a factor of 10^3^ at the time of the patient's first positive PCR test. Cases were categorized into an Omicron group, with respective sub-lineages, and a pre-Omicron group.

Each case was matched with one MRI scan from a non-infected pregnant woman, performed before the SARS-CoV-2 pandemic, for statistical comparison. Criteria for matching were a maximum age difference of ±6 days of gestational age of the fetuses, sex of the fetuses, and field strength of the MR scanner. There were no significant differences between the test subjects and their respective matched control cases with respect to either maternal age (Mean Diff 0.84 years, 95% CI −1.57 to 3.25; *P* = .484; paired t-test), or maternal BMI (Mean Diff 2.26 kg/m^2^, 95% CI −.54 to 5.05; *P* = .110; paired t-test).

Control cases were originally assigned to prenatal MRI with a medical indication. However, only fetuses with no records of clinical conditions that would affect fetal growth, brain development, or placental abnormalities were included as controls (For details of referrals of control cases, see Supplementary Material Table S1). The control cases all had an unremarkable course regarding to time of birth, possible complications during pregnancy, and perinatal outcome.

The institutional review board of the Medical University of Vienna approved this prospective study (Ethics Committee number 2306/2020). Written informed consent was obtained before study inclusion.

### MRI examinations

Morphological signs of placental abnormalities and malperfusion were screened for on fetal MR studies performed on one Philips Ingenia 1.5 Tesla and one Philips Elition X 3 Tesla MRI system at least two weeks after infection with SARS-CoV-2 during pregnancy. The protocol included a minimum of T2-weighted/SSFP sequences in three planes over the fetal brain, body, and placenta, T1 w sequences, and EPI sequences (for detailed MRI acquisition parameters see Supplementary Material Table S2).

All cases with abnormalities in the brain or extracerebral organs received further pre- and postnatal MRIs for further evaluation.

### Image assessment

Placental and fetal structures were examined by a radiologist (D.P.) who specializes in fetal MRI examinations (25+ years of experience in fetal MRI) and was blinded to infected and control cases. To ensure good interrater comparability, another senior (G.K., 15+ years of experience) and junior radiologist (F.P., five years of experience), measured 20 randomly selected cases (10 infected pregnant women and 10 controls) individually and interrater agreement between all three raters was calculated (for details please see Supplementary Material Table S3).

Following the Amsterdam Consensus on gross lesions of MVM,^13^ the placenta was screened for placental hypoplasia or thickening, edema, infarcts, or hemorrhages. Lobules, which can also form to a physiological extent from the 24th week of gestation onward, present with T2-weighted hyperintense bump-like formations with intervening conspicuous septation on MR images and were therefore also expected in the control group.^20^

The shape of the placenta was visually assessed according to the definition of Ohgiya et al.: If a placenta both showed no tapering of placental edges and the placenta was clearly visually thickened, the placenta was qualitatively scored as globoid by the rater.^21^

FGR was assessed either by prenatal ultrasound according to the Delphi consensus (within an average of 6 ± 32 days before/after MRI) or defined as birth weight below the 10th percentile.

Potential lobules and hemorrhages were additionally assessed in terms of their extent as measured by the number of lesions. For this purpose, they were counted on the slice of the insertion of the cord and the adjacent two sliced in each case and categorized (0 lesions: 0 points, 1-2 lesions: 1 point, 3-4 lesions: 2 points, 5+ lesions: 3 points).

For quantitative measurement of the placenta, the slice on which the umbilical cord was attached was identified and the maximum thickness of the placenta was measured on it. For the measurement of the length of the cervix, the slice that best represented its maximum length was used.

In regard to fetal Inflammatory Response Syndrome (FIRS), the thymus, lung maturity, potential inflammatory processes in the respiratory tract, signs of hydrops, and signaling alterations of the intestine and kidneys were screened in detail for abnormalities.

Particular attention was paid to finding possible fetal brain abnormalities and inhomogeneities of the liver on T1 sequences.

### Pathohistological follow-up

Fresh placentas were fixed in 4% buffered formaldehyde. Schematic tissue sampling with six defined locations including umbilical cord removal, fetal membranes, placenta at umbilical cord insertion, peripheral, paracentral, and central, was done with additional samples for macroscopic lesions. Tissue samples were taken at the entire thickness of the placenta including the chorionic and basal plates. Tissue samples were embedded in paraffin blocks, cut into 5 μm sections, and stained with hematoxylin and eosin. Histological assessment was performed by trained placental pathologists with at least 10 years of experience (S.D. & E.L.).

### Statistics

Statistical analysis was performed using SPSS for MacOS (Version 28, IBM, NY/USA). Nominal data were compared using a Chi-square test, and ordinal categorization for determining the extent of hemorrhage/infarction was performed using Wilcoxon signed ranks test. Metric data were compared between infectious disease cases and the 1:1 matched control group using a paired t-test. The Shapiro–Wilks test was used to test for normal distribution. A *P*-value equal to or below .05 was assumed to indicate significant results. Interrater reliability between three raters was calculated using the Interclass Correlation Coefficient for metric parameters and Fleiss Kappa for nominal/ordinal parameters.

## Results

### Study population

Forty-four prenatal MRI examinations were performed between July 2020 and July 2022 in pregnant women after SARS-CoV-2 infection. On average, MRIs were performed 83 days (±42.9, median 80 days) after the first positive PCR test.

**Fig. 1:**
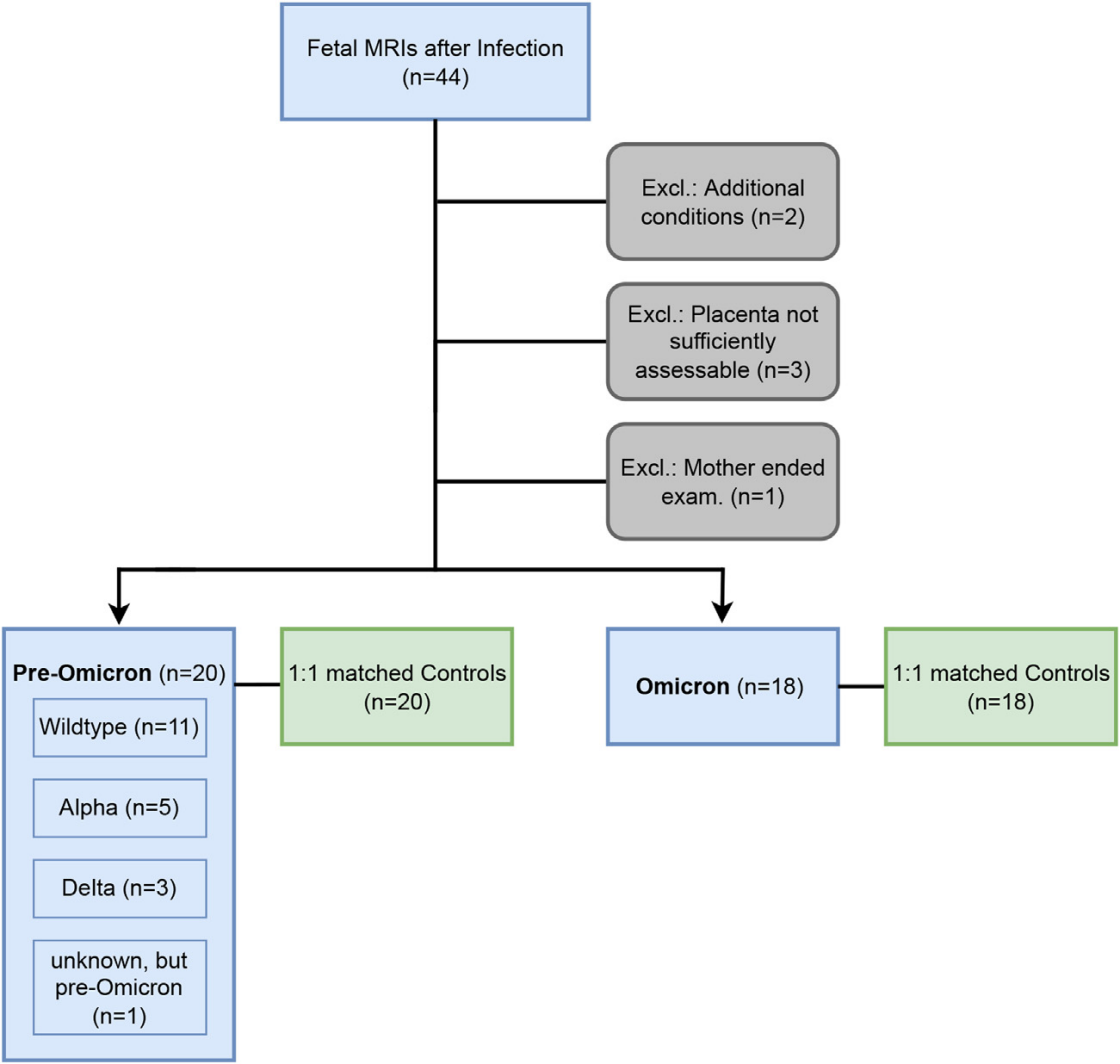
Flow diagram of study subjects.

Two cases had to be excluded because of other detected disease patterns (genetic syndrome, CMV infection during pregnancy) that would have had a potential impact on the fetus and placenta. In three cases insufficient image quality prohibited assessment of the placenta. In another case, the mother discontinued the examination early (Fig. 1).

Twenty infections were pre-Omicron infections with SARS-CoV-2 (wild type n = 11, Alpha [B.1.1.7] n = 5, Delta [B.1.617.2] n = 3). In n = 1, no exact variant could be determined, but they were clearly datable to the pre-Omicron period). Eighteen cases were due to infections with the Omicron variant (B.1.1.529).

The case numbers of the different viral variants in the pre-Omicron group were too small (Alpha n = 5, Delta n = 3) to perform statistically valuable subgroup analyses. Due to a lack of sequencing, the Omicron variants could not be separated into sub-lineages (BA.1–BA.5) (Table 1).

**Table 1:** Characteristics of test subjects and control group.

	Pre-Omicron Group					Pre-Omicron controls	Omicron group	Omicron controls	Total
	SARS-CoV-2 wild-type	Alpha (B.1.1.7)	Delta (B.1.617.2)	Unknown, but pre-Omicron	Totals pre-Omicron		Omicron (B.1.1.529 with respective sub-lineages)		
Cases (n)	11	5	3	1	20	20	18	18	76
Age of pregnant woman *years* ± *SD*	30.12 ± 8.67	35 ± 5.24	35 ± 2.65	33	31.65 ± 5.63	30.70 ± 4.56	31.72 ± 4.47	31 ± 5.58	31.26 ± 5.01
Body mass index *kg/m*^*2*^	29.09 ± 5.61	31.40 ± 11.69	28.05 ± 6.55	27.85	30.02 ± 8.62	27.19 ± 4.55	28.76 ± 6.39	27.15 ± 3.21	28.30 ± 6.09
Male fetuses *n (%)*	8 (72.7%)	1 (20%)	2 (66.7%)	0 (0%)	11 (55%)	11 (55%)	9 (50%)	9 (50%)	40 (52.6%)
Female fetuses *n (%)*	3 (27.3%)	4 (80%)	1 (33.3%)	1 (100%)	9 (45%)	9 (45%)	9 (50%)	9 (50%)	36 (47.4%)
1.5 T MRI *n (%)*	10 (90.9%)	4 (80%)	3 (100%)	1 (100%)	18 (90%)	18 (90%)	17 (94%)	17 (94%)	70 (92.1%)
3 T MRI *n (%)*	1 (9.1%)	1 (20%)	0 (0%)	0 (0%)	2 (10%)	2 (10%)	1 (6%)	1 (6%)	6 (7.9%)
Gestational days at infection *days* ± *SD*	108.18 ± 49.21	137 ± 44.8	88.33 ± 34.56	n/a	112.63 ± 46.90	n/a	132.15 ± 33.08	n/a	n/a
Time difference between infection and MRI *days* ± *SD*	89.91 ± 48.76	53.2 ± 38.13	117.33 ± 63.82	n/a	84.58 ± 50.66	n/a	81.08 ± 29.83	n/a	n/a

On average, MRIs of the sex-matched control cases were performed 1.76 ± 1.65 days (median 1.5 days) later or earlier in pregnancy than in the test subjects.

Including those, a total of 76 prenatal MRIs were evaluated.

Pregnant women were between gestational week (GW) 19 + 2 and 36 + 6 at the time of MRI examination, with an average GW of 28 + 4 ± 4.4 (median GW 27 + 7). Seventy examinations were performed on a 1.5 Tesla Philips Ingenia and six on a 3 Tesla Philips Elition X.

One patient with the Omicron variant required hospitalization (this patient showed a conspicuous globular placenta with lobulations). No patient had to be admitted to the intensive care unit.

### Changes in placentas after infection with SARS-CoV-2

Placental changes presented as globular shapes in pre-Omicron-variants (30% [n = 6] vs. 0% in the control group) and the Omicron group (27.8% [n = 5] vs. 0%).

As for lobulation and hemorrhages in the placentas, both groups showed an increased incidence after SARS-CoV-2 infection (see Table 2). Still, the Chi-square test was significant only for the variants in pre-Omicron variants for lobulation (*P* = .046) and hemorrhages (*P* = .002).

**Table 2:** Overview of detected abnormalities.

	pre-Omicron	pre-Omicron-controls	*P*-values pre-Omicron	Omicron	Omicron-controls	*P*-values Omicron
n	20 (100%)	20 (100%)		18 (100%)	18 (100%)	
Globular shape^a^	6 (30%)	0 (0%)	**.008**	5 (27.8%)	0 (0%)	**.016**
Lobules^a^	13 (65%)	8 (40%)	**.046**	11 (64.7%)	8 (44.4%)	.229
Hemorrhages^a^	11 (55%)	2 (10%)	**.002**	5 (27.8%)	4 (22.2%)	.700
FGR^a^	5 (25%)	0 (0%)	**.017**	1 (5.6%)	0 (0%)	.310
Extent of lobulation^b^			**.035**			**.710**
Extent of hemorrhage^b^			**.012**			**.739**
Thickness of placenta^c^			**.048 (6.35, 95% CI .02–12.65)**			.214 (5.28, 95% CI −3.35 to 13.91)
Lengths of cervix^c^			.579 (1.94, 95% CI −5.32 to 9.21)			.736 (–.65, 95% CI −4.65 to 3.36)

Bold = A P-value equal to or below .05 was assumed to indicate significant results.

a n (%) of abnormalities and *P*-values for Chi-square by appearance compared to the respective control group.

b *P*-values for the categorized extent of placental changes compared to the respective control group (Wilcoxon signed ranks test).

c *P*-values for differences in measured lengths (Paired t-test with 1:1 matched control cases; [Mean Diff in mm, 95% CI]).

Both extents of lobulation and hemorrhages compared with the respective control cases were significant in the pre-Omicron cases (lobulation *P* = .035; hemorrhages *P* = .012).

Significantly evident (Mean Diff (6.35, 95% CI .02–12.65), *P* = .048), was the increase in the thickness of placentas in the pre-Omicron variants (Table 2).

No significant differences were seen regarding the length of the cervix.

Two unvaccinated patients in the Omicron group showed abnormal globular placentas. There were 0 globular placentas in 1x-vaccinated (n = 2), one in 2x-vaccinated (n = 4), and one in 3x-vaccinated (n = 6). No vaccination status was elicitable from the remaining four pregnant women (one globular placenta) in the Omicron group. Due to the limited availability during the emergence of the pre-Omicron variants, no conclusions were made regarding vaccination status in this group.

Interrater agreement between the rater, another senior radiologist and one junior radiologist was good to excellent regarding the measurement of placental thickness, cervical lengths, and assessment of placental shape, lobulation, and hemorrhages (see Supplementary Material Table S3).

**Fig. 2:**
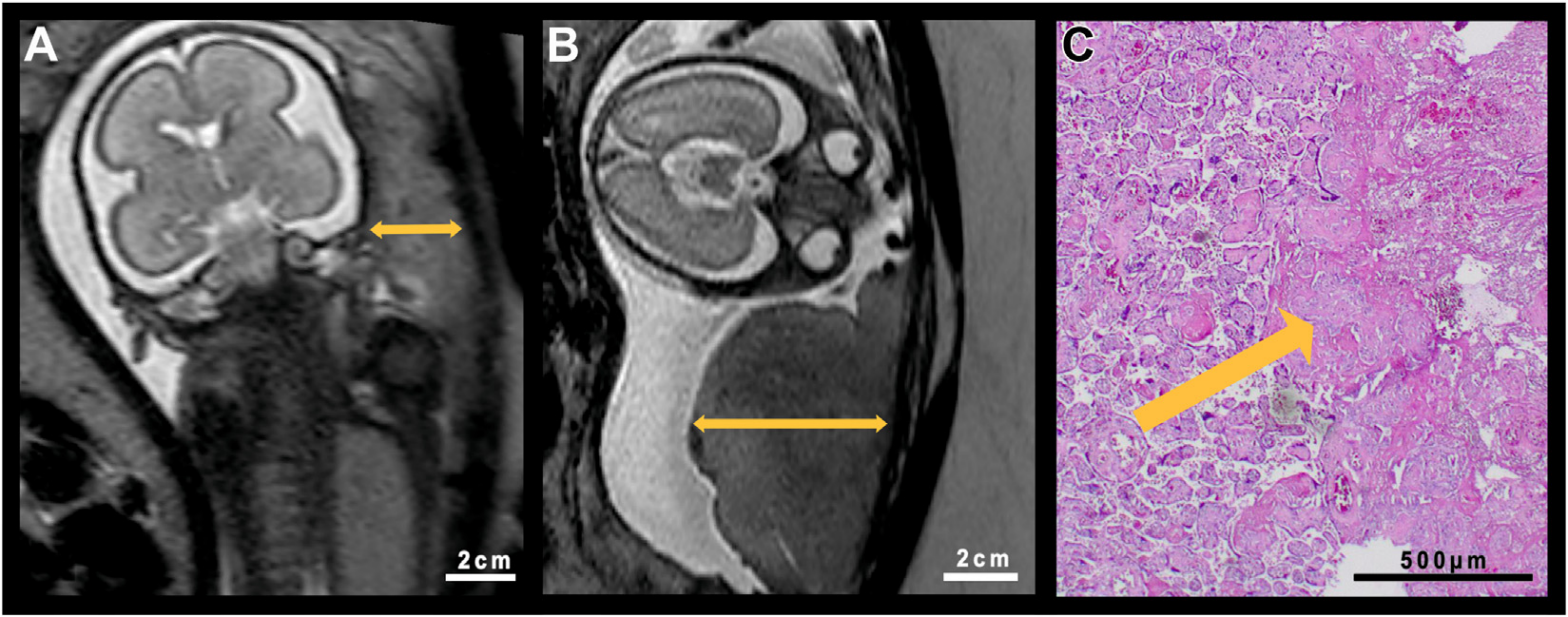
Comparison of a globular placenta and its control case. A. Age-appropriate placenta of a healthy control fetus at GW 25 + 1 in a T2-weighted MRI sequence. B. Edematous/globular thickened placenta of a fetus with FGR at GW 24 + 5 after SARS-CoV-2 infection of the pregnant woman. T2-weighted sequence. C. Postnatal histological correlate of the placenta of the fetus of image B. Arrow points towards an area of infarction.

Histopathologic studies of the placentas were available in 11 (55%) pre-Omicron cases. One of these (9%) showed no pathological changes in histology or MRI. The other 10 (91%) placentas were histologically conspicuous: three (27%) showed inflammatory processes; seven (64%) intervillous fibrin deposits; four (36%) thrombosed areas; and six (55%) infarcted lesions. Histological examination of one placenta was available in the Omicron group, showing fibrin deposits and infarcted areas (Fig. 2).

Cases of FGR were observed in 25% (n = 5, pre-Omicron), and 5.6% (n = 1, Omicron). In the pre-Omicron group, cases with FGR showed significant correlations with globular (*P* = .002) placental expressions.

### Fetal changes

**Fig. 3:**
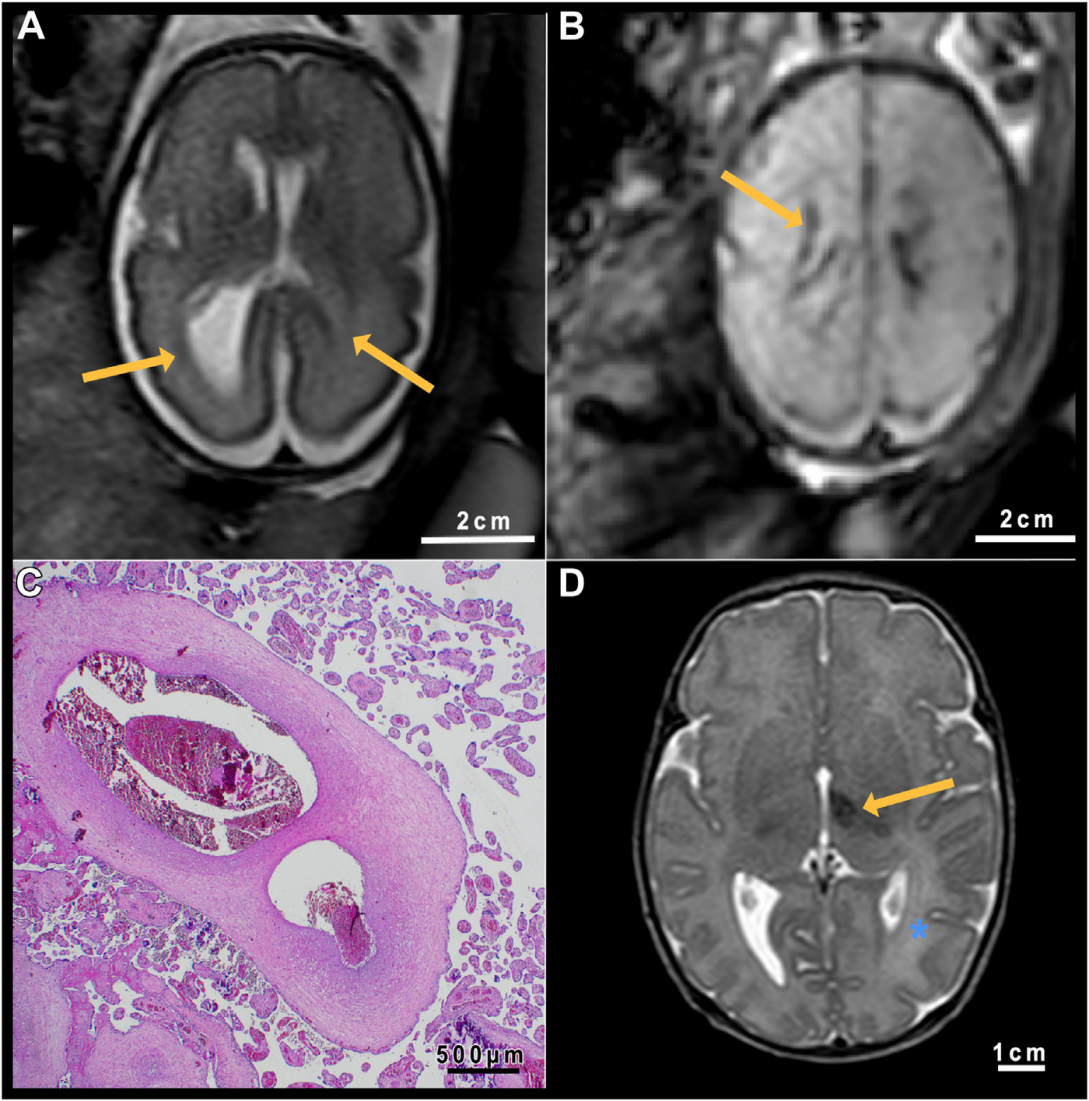
Case of infection with SARS-CoV-2 wild type. A. T2-weighted fetal MRI image at GW 27 + 1 with unilateral ventriculomegaly (arrows) Section at basal ganglion level. B. Blood-sensitive sequence in the same MRI session. The arrow points toward the hemorrhagic deposit of the ventricular wall. Note the placental stasis hemorrhage. Level at semioval center. C. Histological examination of the placenta in this case: Ectatic vessels with the presence of older, organized thrombi. D. T2-weighted sequence of the brain at seven days postpartum. The orange arrow points to a hemorrhagic lesion in the left thalamus. The blue star marks a perinatal anatomical-left parieto-occipital infarction, characterized by hyperintensity of the white matter.

Perinatal cerebrovascular fetal lesions occurred in two fetuses after infection (both wild type). The cases showed hemorrhages along the ventricular wall and asymmetric ventricle configuration (see Fig. 3). In both cases, the placentas were conspicuous for the shape or lobulations and intraplacental hemorrhages.

Postnatal MRI was available in both cases. One child also showed a perinatal new-onset left media infarct and a hemorrhagic lesion in the left thalamus.

Additional nonspecific signal hypointensities in T1-weighted sequences were seen in the liver of two fetuses. In all these cases, placental changes were detected. Respiratory tract, thymus, kidneys, and intestine were unsuspicious in all MRI scans.

Of the Covid cases, pregnancy outcome data were available in 30 of 38 (79%) cases with infections. Preeclampsia occurred in one case (Omicron variant), one patient (infection with wild type) underwent C-section in GW 36 + 0 due to massive FGR, and one patient (infected with Alpha variant) decided to terminate her pregnancy in GW 19 + 5 due to severe FGR. In these three cases, the placentas were highly abnormal in terms of shape, lobulation, and hemorrhages.

All other fetuses were term born.

## Discussion

The main finding of this study is that MRI detects placental damage by SARS-CoV-2 infection. Importantly, pre-Omicron subtypes had a more severe effect than Omicron sub-lineages on placental function.

We demonstrated globular shape changes of the placenta in infections with the Omicron variant (*P* = .016) and in infections with previously circulating SARS-CoV-2 variants compared to non-infected controls (*P* = .008). The pre-Omicron variants showed additional increased frequency in lobulation (*P* = .046), hemorrhages (*P* = .002), and significance in the thickening of the placenta (6.35, 95% CI .02–12.65, *P* = .048). This thickening with subsequent globular shape changes appears to be a compensatory mechanism in placental insufficiency, also known to be associated with vascular causes.^22^ Thus, the presented results provide evidence that the effects on the fetus are caused by malperfusion of placental vessels.

Histologic correlates also support vascular genesis. Evidence for perfusion defects points toward fetal vascular malperfusion, as indicated by the infarcted areas and the partially thickened vessel walls, which probably developed based on thrombi (see Fig. 2, Fig. 3). The detected intervillous thrombi and the intervillous fibrin deposits indicate MVM. The association between an increased risk of thromboembolic events and coagulopathies with COVID-19 is already well established, with an increased risk in relation to disease severity.^23^^,^^24^ The risk of thromboembolic events may differ for the respective variants of SARS-CoV-2, but data on the recent Omicron variants are lacking. The pathophysiology is likely multifactorial and results from an interplay of immune-inflammatory response and endothelial injuries leading to microvascular thromboses.^25^

Endothelial injury parallels adult lung tissue, where, as in the placentas, high levels of the expressed integral membrane protein ACE2 are found.^6^^,^^26^ These endothelial lesions in the form of disrupted cell membranes in the adult vascular system were observed with an accumulation of inflammatory cells and detectable viral components after SARS-CoV-2 infection.^26^ In this context, the different SARS-CoV-2 variants have different pathogenic impacts.

Compared to the described pathological changes in placentas, thrombi, infarcted areas, hemorrhages, and the formation of lobules are also known to occur to a smaller extent during normal “placental aging” also in non-infected pregnancies—these changes are usually not seen before the 24th week of gestation on fetal MRI and show a correlate in postnatally histopathological examinations.^27^^,^^28^

The increased incidence of FGR after infection with SARS-CoV-2 observed in this study is in accordance with a placental insufficiency-related cause. Stoecklein et al. showed that fetal lung volume is reduced after SARS-CoV-2 infection, which is also well consistent with the FGR and vascular placental insufficiencies we detected.^29^

The high susceptibility of the placenta to SARS-CoV-2 could be explained due to the virus entering the cells by docking to the angiotensin-converting enzyme 2 (ACE2), highly expressed in fetal placental vessels, stromal cells of the decidua, and in intestinal tract, or ovaries.^6^^,^^30^ Furthermore, the virus utilizes the serine protease TMPRSS2 for spike protein priming.^30^ The co-expression of ACE2 and TMPRSS2 in placental cells may facilitates SARS-CoV-2 cellular entry.^6^ The placenta is likely to provide an effective immunologic barrier to SARS-CoV-2 and protect fetuses from direct exposure to the virus, as shown by the low vertical transmission rates.^3^, ^4^, ^5^

This may be at least partially explained by the presence of a large number of immune cells in the placental tissue: In the gestational decidua, large numbers of macrophages, natural killer (NK) cells, and dendritic cells infiltrate the decidua during the first trimester and cluster around the invading trophoblast cells.^31^

Three fetuses showed organ abnormalities (one with vascular brain lesions, one with nonspecific signal alterations in the liver, and one with a brain lesion and liver changes).

In the cases with prenatal brain abnormalities, the synopsis with perinatal MRI examinations is noteworthy, also showing the possibility of further perinatal vascular events in the brain and emphasizing the pathophysiological vascular component of SARS-CoV-2 infections during pregnancy. Mappa et al. showed in a biometry of fetal brains after SARS-CoV-2 infection using ultrasound that general brain growth and cortical development were not affected.^32^ However, our findings on abnormalities in the fetal brain are based on vascular events. The latter is particularly interesting when considering the interplay in the excretion of fetally produced cholephilic organic anions, which are potentially toxic, and in the elimination of which both placenta and fetal liver play an essential role.^33^

In our study, the fetuses in the group with pre-Omicron variants showed more frequent placental lesions in terms of lobulation and hemorrhages than in the Omicron group. The extent of lobulation and hemorrhages compared to the respective control cases was, however, significant in pre-Omicron and Omicron group. Globular shape changes occurred in both groups. These results are consistent with the recent histopathologic study by Shanes et al.: Analysis of 883 placentas after SARS-CoV-2 infection during pregnancy and 185 control cases showed that the different viral variants each exert a distinct influence on the pathologic changes in the placenta.^10^ Histopathologic changes can be detected after SARS-CoV-2 infections in all trimesters of pregnancy. MVM features were more common in infections with the Delta variant compared to Alpha or Gamma variant and even less common with Omicron.

This varying occurrence of placental lesions due to different viral variants may be due to the lower pathogenicity of the Omicron variant and to the higher vaccination coverage rate as the pandemic progressed. Our results show that the two unvaccinated pregnant women infected with SARS-CoV-2-Omicron developed globular placentas, but only one of six triple-vaccinated women did.

Pregnant women after COVID-19 infection should be screened for placenta insufficiency using prenatal ultrasound or other forms of fetal imaging including a close morphological examination of the placenta. To date, it remains unclear to what extent the lesions found in our MRI study after infection with SARS-CoV-2 could also be detected by ultrasound.

Using MRI, a very good-to-excellent interrater reliability was shown. Thus, prenatal MRI can be a reliable *in vivo* diagnostic tool for potential SARS-CoV-2-associated pathologies of the fetus and placenta.

### Strengths and limitations

One strength of this study lies in providing evidence that SARS-CoV-2-associated placental changes occur early in pregnancy. Although our study design does not allow for a definite differentiation between possible effects by vaccination or the pathogenicity of Omicron sub-lineages it emphasizes, the substantial impact of pre-Omicron variants on early placental development. This was further proven by pathohistological confirmation in a subset of cases. By retrospectively including MRIs of non-infected pregnant women before the pandemic as “historic” controls, we could exclude any effect of SARS-CoV-2 on placental growth in our control group. Unfortunately, it has not been possible to provide enough cases for a detailed statistical comparison of each virus variant. The reliability of a fully automated quantification of placental alterations by texture analysis or placental MR signal quantification is currently limited. Thus, we have decided to perform a radiological expert-based semi-quantitative assessment provided by experienced fetal radiologists with a good interrater agreement. Finally, information on preexisting maternal diseases and risk factors were not available in all cases. However, we were careful not to include any case with disease or obstetric events that would have had an impact on the parameters we measured.

### Conclusion

In this case–control study, prenatal MRI after SARS-CoV-2 infection in pregnancy reveals placental lesions based on vascular malperfusion. These changes are more pronounced in pre-Omicron variants than in Omicron and provide an explanation for SARS-CoV-2-associated morbidity of the fetus, such as fetal growth restriction.

## Contributors

Patric Kienast: data analysis, data curation, statistics, writing original draft.

Daniela Prayer: conceptualisation, study design, data collection, data analysis, data interpretation, validation.

Julia Binder: study design, data collection.

Florian Prayer: data collection, data analysis.

Sabine Dekan: data collection, data analysis, methodology.

Eva Langthaler: data collection, data analysis.

Benjamin Sigl: data analysis, validation.

Sabine Eichinger: supervision.

Nicole Perkman-Nagele: supervision.

Ingrid Stuempflen: resources, project administration.

Marlene Stuempflen: data curation.

Nawa Schirwani: data collection.

Petra Pateisky: data collection.

Christian Mitter: data collection, validation.

Gregor Kasprian: funding acquisition, investigation, supervision, validation.

All authors: writing-review and editing.

## Declaration of interests

The authors declare no conflict of interest.
