# Supplementary material for: SARS-CoV-2 variant-related abnormalities detected by prenatal MRI: a prospective case–control study
Source: Lancet Reg Health Eur. 2023 Jan 21;26:100587. doi: 10.1016/j.lanepe.2023.100587 (PMC9860502; doi:10.1016/j.lanepe.2023.100587)
Supplement: Supplementary Tables S1–S3 [file mmc1.docx]

SARS-CoV-2 variant-related abnormalities detected by prenatal MRI: A prospective case-control study

Patric Kienast, MD^1^; Daniela Prayer, MD^1^; Julia Binder, MD, PhD^2^; Florian Prayer, MD, PhD^1^; Sabine Dekan, MD^3^; Eva Langthaler, MD^3^; Benjamin Sigl, MD^1^; Sabine Eichinger, MD^4^; Nicole Perkmann-Nagele, MD^5^; Ingrid Stuempflen, BSc^6^; Marlene Stuempflen, MD^1^; Nawa Schirwani, MD^2^; Petra Pateisky, MD^2^; Christian Mitter, MD^1^; Gregor Kasprian, MD^1^

^1^ Department of Biomedical Imaging and Image-Guided Therapy, Division of Neuroradiology and Musculoskeletal Radiology, Medical University of Vienna, Vienna, Austria

^2^ Department of Obstetrics and Feto-Maternal Medicine, Medical University of Vienna, Vienna, Austria

^3^ Department of Pathology, Medical University of Vienna, Vienna, Austria

^4^ Department of Medicine I, Division of Hematology and Hemostaseology, Medical University of Vienna, Vienna, Austria

^5^ Department of Laboratory Medicine, Medical University of Vienna, Vienna, Austria

^6^ Department of Obstetrics & Gynecology, Klinik Floridsdorf, Vienna, Austria

### Corresponding author

Assoc. Prof. Priv.-Doz. **Gregor Kasprian**, MD
**Eml.** [gregor.kasprian@meduniwien.ac.at](mailto:gregor.kasprian@meduniwien.ac.at)
**Tel.** +43-1-40400 – 48180
**Adr.** Universitätsklinik für Radiologie und Nuklearmedizin, Währinger Gürtel 18-20, 1090 Wien, Austria

## Supplementary Material

**Table 1: Referral diagnoses of control cases made by ultrasound**

| Suspicious diagnosis in ultrasound | n (%) | Prenatal MRI diagnosis |
| --- | --- | --- |
| No detected abnormalities on US, but complex malformation syndromes known in siblings | 14 (36·84 %) | No detected abnormalities in fetal or extrafetal structures |
| No detected abnormalities on US, but maternal gestation diabetes in combination with previous aborts | 5 (13·16 %) | No detected abnormalities in fetal or extrafetal structures |
| Intestinal tract stenosis | 3 (7·89 %) | Disproved suspected US diagnosis. No detected abnormalities in fetal or extrafetal structures |
| Anal atresia | 1 (2·63 %) | Disproved suspected US diagnosis. No detected abnormalities in fetal or extrafetal structures |
| Cleft lip and palate | 1 (2·63 %) | Disproved suspected US diagnosis. No detected abnormalities in fetal or extrafetal structures |
| Mega cisterna magna | 1 (2·63 %) | Disproved suspected US diagnosis. No detected abnormalities in fetal or extrafetal structures |
| Cavum septi pellucidi et vergae | 1 (2·63 %) | Cavum septi pellucidi et vergae as norm variant |
| Minimal lateral ventricular asymmetry (<2mm) | 5 (13·16 %) | Isolated ventricle asymmetry with ventricular width in normal range bilaterally |
| Hydronephrosis | 3 (7·89 %) | Confirmed diagnosis; no effect on fetal growth; no abnormalities in other fetal organs or placenta. |
| Polycystic kidney disease | 4 (10·53 %) | Confirmed diagnosis; no effect on fetal growth; no abnormalities in other fetal organs or placenta. |

**Table 2: Acquisition parameters of used sequences in fetal MRI.**

|  |  | Scanner | |
| --- | --- | --- | --- |
|  |  | 1·5-T Ingenia (Philips Medical Systems, the Netherlands) with a 32-channel Multi Coil | 3.0-T Elition X (Philips) with a 32-channel Abdomen Coil |
| Sequences | Steady-state free precision (SSFP) | Slice thickness 3-6 mm, slice gap 50%, field of view (FOV) 256×256mm, matrix 188×214, echo time (TE) 1·78ms, repetition time (TR) TE-dependent, flip angle 80° | Slice thickness 3-6mm, slice gap 50%, FOV 336x336mm, matrix 200x176, TE 1·9ms, TR: TE-dependent, flip angle 90° |
|  | T2-weighted turbo spin echo (TSE). Head-sequences | Slice thickness 3mm, slice gap 50%, FOV 336×336mm, matrix 260×172, TE 140ms, TR: TE-dependent, flip angle 90° | Slice thickness 2·5mm, slice gap 50%, FOV 240×240mm, matrix 228×205, TE 200ms, TR: TE-dependent, flip angle 90° |
|  | T2-weighted turbo spin echo (TSE) Abdominal-sequences | Slice thickness 4mm, slice gap 50%, FOV 320×320mm, matrix 256×166, TE 100ms, TR: TE-dependent, flip angle 90° | Slice thickness 3mm, slice gap 50%, FOV 288×288mm, matrix 272×243, TE 120ms, TR: TE-dependent, flip angle 90° |
|  | T1-weighted Fast Field Echo (T1-FFE) | Slice thickness 4mm, slice gap 50%, FOV 288x288mm, matrix 208x167, TE 4·6ms, TR: TE-dependent flip angle 50° | Slice thickness 4mm, slice gap 50%, FOV 224x224mm, matrix 163x82, TE 2·41ms, TR: TE-dependent, flip angle 15° |
|  | Echo-planar imaging (EPI) | Slice thickness 3mm, slice gap 50%, FOV 256x256mm, matrix 124x125, TE 53·5ms, TR: TE-dependent, flip angle 90° | Slice thickness 2mm, slice gap 50%, FOV 176x176mm, matrix 128x123, TE 47·08ms, TR: TE-dependent, flip angle 90° |

**Table 3: Interrater reliability between two senior and one junior radiologist.**

| Parameter | Interclass Correlation Coefficient (metric parameters, two-way mixed models, absolute agreement. average measures) | Fleiss Kappa (ordinal parameters, overall agreement) |
| --- | --- | --- |
| Thickness of placenta | ·926 |  |
| Length of cervix | ·955 |  |
| Placental shape |  | 1 |
| Extent of lobulation |  | ·738 |
| Extent of hemorrhages |  | ·653 |
| Detected brain abnormalities |  | 1 |
| Detected liver abnormalities |  | 1 |
